# Musculoskeletal health from the “One Medicine” perspective – what can we learn from large and small animal models (with emphasis on articular cartilage)?

**DOI:** 10.1186/1471-2474-16-S1-S6

**Published:** 2015-12-01

**Authors:** P René van Weeren, Marianna A Tryfonidou

**Affiliations:** 1 Department of Equine Sciences, Faculty of Veterinary Medicine, Utrecht University, Yalelaan 112, 3584CM Utrecht, the Netherlands; 2 Department of Clinical Sciences of Companion Animals, Faculty of Veterinary Medicine, Yalelaan 108, 3584CM, Utrecht, Utrecht University, the Netherlands

## 

In human medicine musculoskeletal diseases rank (together with mental disorders) first in reasons for occupational disability and have a huge impact on both quality of life and overall healthcare costs[1]. The current increase in life expectancy, together with decreasing societal acceptance of impaired mobility, have strongly pushed musculoskeletal research in recent years.

The classic animal models for research into musculoskeletal disease are small rodents, especially mice and rats. As larger species, goats and to a lesser extent sheep have been the species of choice. This choice was largely based on practical and logistical considerations such as the required size, availability, costs and ease of handling, rather than on biomedical criteria.

The growing acceptance of the “One Health, One Medicine” concept has, together with better knowledge of fundamental differences between mammalian species in articular cartilage biology and the increasing pressure to reduce, refine and replace (the three “Rs”) animal experimentation, led to a change in attitude towards the use of animal models in musculoskeletal research[2]. Whereas small rodents may still be a logical step after *in vitro* research, the fundamental differences between articular cartilage composition of smaller species and those heavier than about 1Kg[3], together with the increasing recognition of the role of biomechanics within the joint, cast severe doubts on the validity of these species for anything but very basic work in musculoskeletal research. In contrast, within the “One Medicine” concept it is clear that in veterinary medicine there are several species featuring a high prevalence of musculoskeletal disorders that are very similar to those seen in humans. This applies to dogs with intervertebral disc disease[4] and chronic joint disorders (especially osteoarthritis (OA)) in both horses and dogs[4]. These developments have led to a gradual shift in the use of animals in musculoskeletal research. Also, regulatory bodies are making this shift of mind with the US Food and Drug Administration (FDA) now requiring preparatory work in horses before approval for certain orthopaedic devices is granted.

There is one other important aspect to this development. Whereas the classic animal models were solely used to the benefit of human research, research in dogs and horses will forcibly lead to medical improvements for these species, as they are patients too and hence not only experimental animals, but target species as well. This is an important asset for the ethical justification for the use of animals for scientific research.
